# ADAM12 and ADAM17 are essential molecules for hypoxia-induced impairment of neural vascular barrier function

**DOI:** 10.1038/srep12796

**Published:** 2015-08-05

**Authors:** Dan Cui, Mitsuru Arima, Keiyo Takubo, Tokuhiro Kimura, Keisuke Horiuchi, Takuya Minagawa, Satoshi Matsuda, Eiji Ikeda

**Affiliations:** 1 Department of Pathology, Yamaguchi University Graduate School of Medicine, 1-1-1 Minami-Kogushi, Ube, Yamaguchi 755-8505, Japan; 2 Department of Ophthalmology, Kyushu University Graduate School of Medical Sciences, 3-1-1 Maidashi, Higashi-ku, Fukuoka City, Fukuoka 812-8582, Japan; 3 Department of Stem Cell Biology, Research Institute, National Center for Global Health and Medicine, 1-21-1 Toyama, Shinjuku-ku, Tokyo 162-8655, Japan; 4 Departments of Cell Differentiation, The Sakaguchi Laboratory of Developmental Biology Keio University School of Medicine, 35 Shinanomachi, Shinjuku-ku, Tokyo 160-8582, Japan; 5 Department of Orthopedic Surgery, Keio University School of Medicine, 35 Shinanomachi, Shinjuku-ku, Tokyo 160-8582, Japan; 6 Department of Surgery, Keio University School of Medicine, 35 Shinanomachi, Shinjuku-ku, Tokyo 160-8582, Japan; 7 Department of Cell Signaling, Institute of Biomedical Sciences, Kansai Medical University, 10-15 Fumizono-cho, Moriguchi, Osaka 570-8506, Japan

## Abstract

Neural vascular barrier is essential for the life of multicellular organisms, and its impairment by tissue hypoxia is known to be a central of pathophysiology accelerating the progression of various intractable neural diseases. Therefore, the molecules involved in hypoxia-induced impairment of vascular barrier can be the targets to establish new therapies for intractable diseases. Here, we demonstrate that a disintegrin and metalloproteinases (ADAMs) 12 and 17 expressed in endothelial cells are the molecules responsible for the impairment of neural vascular barrier by hypoxia. Brain microvascular endothelial cells *in vitro* lost their barrier properties immediately after hypoxic stimulation through diminished localization of claudin-5, a tight junction molecule, on cell membranes. Hypoxic disappearance of claudin-5 from cell membranes and the consequent loss of barrier properties were completely suppressed by inhibition of the metalloproteinase activity which was found to be attributed to ADAM12 and ADAM17. Inhibition of either ADAM12 or ADAM17 was sufficient to rescue the *in vivo* neural vasculature under hypoxia from the loss of barrier function. This is the first report to specify the molecules which are responsible for hypoxia-induced impairment of neural vascular barrier and furthermore can be the targets of new therapeutic strategies for intractable neural diseases.

Blood vessels of neural tissues constitute the physical and biochemical barriers such as the blood-brain barrier and the inner blood-retinal barrier. These neural vascular barriers are essential for the life of multicellular organisms through homeostatic regulation of tissue microenvironment upon which the function of neural cells depends. Neural vascular barriers are induced during the embryonic development, but the once established barriers in adult are still under the dynamic regulation in response to tissue oxygen concentration, inflammatory cytokines and so forth^1^,^2^,^3^,^4^,^5^. Among these triggers, the decrease in tissue oxygen concentration, tissue hypoxia, is known to be a major trigger to impair the vascular barrier in various pathological situations of neural tissues, and hypoxia-induced impairment of vascular barrier function works as a core pathological factor to accelerate the progression of intractable neural diseases including diabetic retinopathy and ischemic cerebral attack^1^,^6^,^7^. However, the mechanisms how tissue hypoxia opens neural vascular barrier remain unclarified.

Neural vascular barrier function is attributable mainly to the complex tight junction (TJ) strands formed between endothelial cells. TJs are composed of membrane spanning molecules, such as occludin, claudins consisting of 27 family members and junctional adhesion molecule (JAM), which interact with cytoplasmic proteins, ZO-1, ZO-2 and ZO-3^8^. Our previous study has demonstrated that hypoxia disrupts the neural vascular barrier by decreasing the protein level of claudin-5, a member of claudin family, on cell membranes of endothelial cells^9^. Therefore, in order to specify the molecules which play the essential role in the impairment of neural vascular barrier by hypoxia, we have focused our study on the mechanisms of hypoxia-triggered changes in claudin-5 expression. Although several molecules such as caveolin-1, caspases, matrix metalloproteinases (MMPs), ADAMs as well as molecules in ubiquitin-proteasomal system are reported to be involved in the processing of TJ molecules, the mechanisms of oxygen concentration-dependent regulation of claudin-5 expression remain unknown^10^,^11^,^12^,^13^,^14^.

## Results

### Enhanced disappearance of claudin-5 from endothelial cell membranes under hypoxia, in parallel with the loss of barrier property

**Figure 1:**
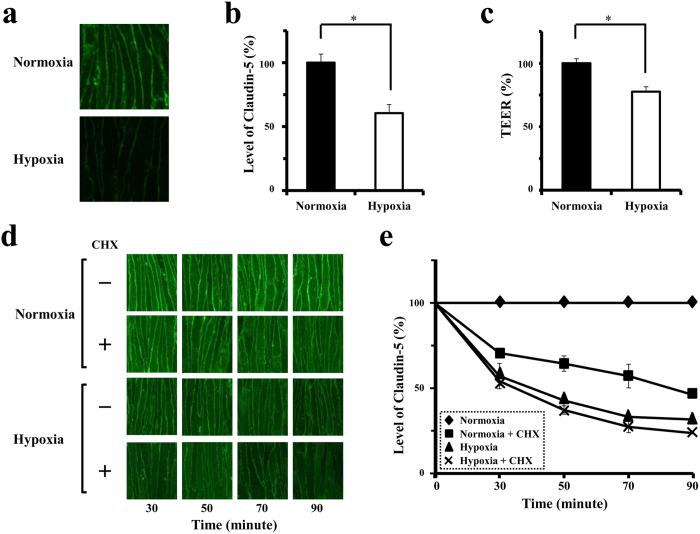
Hypoxia accelerates the disappearance of claudin-5 from cell membranes, and consequently impairs the barrier property of endothelial cells. (**a**) Immunofluorescence images for claudin-5 expression in bEnd.3 monolayers under normoxia or hypoxia for 30 minutes. (**b**) Quantitative analysis of claudin-5 levels on cell membranes corresponding to the images in a. (**c**) TEERs of bEnd.3 monolayers under normoxia or hypoxia. Levels of claudin-5 protein on cell membranes as well as the TEERs of bEnd.3 monolayers are decreased in response to hypoxia. (**d** and **e**) Confocal scanning microscopic images (**d**) and their corresponding quantitative analysis of claudin-5 levels on cell membranes of bEnd.3 cells under normoxia or hypoxia for 30, 50, 70 and 90 minutes after pretreatment with or without CHX. Although claudin-5 disappeared from cell membranes quickly even under normoxia, the disappearance of claudin-5 is shown to be accelerated under hypoxia. Data are presented as mean ± SD from 3 independent cultures. *P < 0.05.

Monolayers of bEnd.3, mouse brain microvascular endothelial cells, were cultured under normoxia and hypoxia, 21% O_2_ (atmospheric air) and 1% O_2_, respectively. Confocal imaging experiments with quantitative analysis demonstrated that claudin-5 molecules locate on cell membranes adjacent to neighboring cells under normoxia, and that the levels of claudin-5 on cell membranes significantly decrease, in parallel with a fall in the transendothelial electrical resistance (TEER) of cell monolayer, after exposure to hypoxia for 30 minutes (Fig. 1a–c). To monitor the turnover of claudin-5 molecules, the protein levels of claudin-5 on cell membranes were quantitatively analyzed in cells under normoxia or hypoxia for 30, 50, 70 and 90 minutes in the presence or absence of cycloheximide (CHX), a protein synthesis inhibitor. As demonstrated in Fig. 1d,e, the levels of claudin-5 on cell membranes of normoxic cells without CHX treatment were unchanged, while those of normoxic cells with CHX treatment decreased significantly already in 30 minutes and reached around 64.4 ± 2.2% (mean ± SD) of the control in 50 minutes, indicating the rapid turnover of claudin-5 under physiological condition. When the cells are exposure to hypoxia in the presence of CHX, claudin-5 disappeared from cell membranes more rapidly than under normoxia, and reached 37.2 ± 2.5% of the control in 50 minutes. Statistically, hypoxia accelerates the loss of claudin-5 from cell membranes.

### Oxygen concentration-independent and dependent turnovers of claudin-5 on endothelial cell membranes

**Figure 2:**
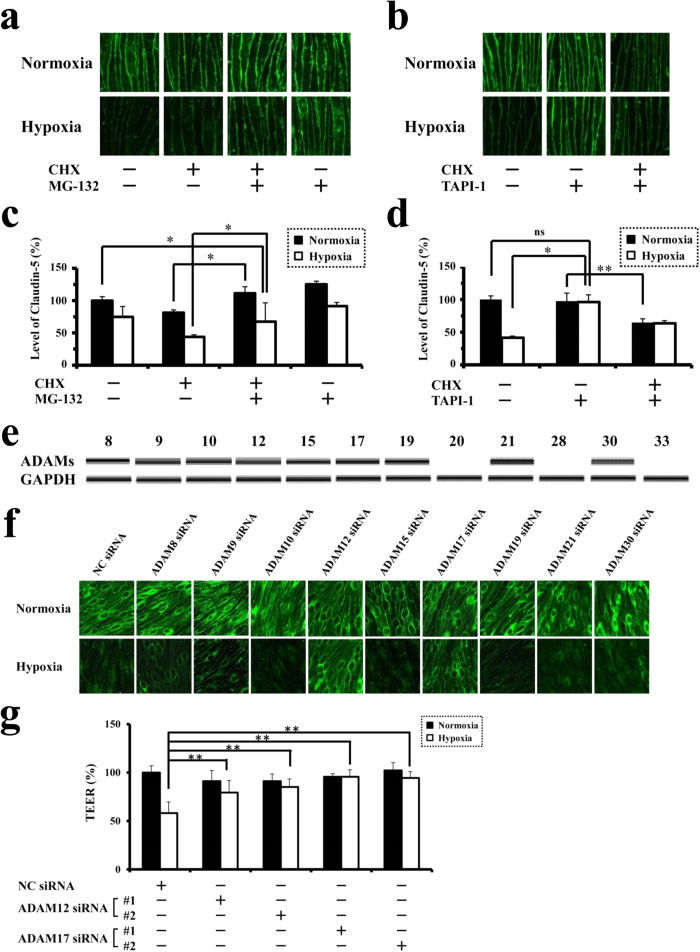
Involvement of ADAM12 and ADAM17 in hypoxia-induced disruption of neural vascular barrier. (**a**) Immunofluorescence images for claudin-5 expression in CHX-untreated or treated bEnd.3 monolayers in the presence or absence of MG-132. (**b**) Immunofluorescence images for claudin-5 expression in CHX-untreated or treated bEnd.3 monolayers in the presence or absence of TAPI-1. (**c**) Quantitative analysis of claudin-5 levels on cell membranes corresponding to the images in a. (**d**) Quantitative analysis of claudin-5 levels on cell membranes corresponding to the images in b. (**e**) RT-PCR for the expression of ADAM family members in bEnd.3 cells under normoxia. (**f**) Immunofluorescence images for claudin-5 of normoxic or hypoxic bEnd.3 monolayers pretreated with the siRNA specific for each member of ADAM family. Two kinds of siRNAs were designed to suppress the expression of each member, and the representative photographs are presented. Hypoxia-induced disappearance of claudin-5 from cell membranes is inhibited with the pretreatment of siRNAs for ADAM12 or ADAM17. (**g**) TEERs of bEnd.3 monolayers under normoxia or hypoxia after the induction of siRNAs for ADAM12 or ADAM17, showing that the suppression either ADAM12 or ADAM17 rescues bEnd.3 monolayers from the impairment of barrier properties under hypoxia. *P < 0.01; **P < 0.05; ns, not significant. NC siRNA; non-silencing siRNA for negative control.

Under normoxic condition, the presence of MG-132, an inhibitor of ubiquitin-proteasome system, suppressed the disappearance of claudin-5 from cell membranes in CHX-treated cells completely to the level of normoxic cells without CHX treatment (Fig. 2a,c), which is consistent with the results of Mandel I *et al.* with HeLa cells^14^. On the other hand, under hypoxic condition, MG-132 treatment rescued CHX-treated hypoxic cells from the disappearance of claudin-5 only partially, not to the level of CHX-untreated normoxic cells (Fig. 2a,c). These data indicate that the physiological turnover of claudin-5 under normoxia is ubiquitin-proteasome system-dependent, while other additional mechanisms must be activated under hypoxia to accelerate the turnover of claudin-5. Based on our previous data showing the post-transcriptional regulation of claudin-5 expression under hypoxia^9^, we hypothesized that some proteases which could process claudin-5 molecules within 30 minutes after hypoxic stimulation accelerate the hypoxic disappearance of claudin-5. ADAMs were thought to be the candidates for responsible proteases, since they are known to be the modulators for processing of various membrane molecules in a manner of quick response to stimuli^15^,^16^,^17^. As shown in Fig. 2b,d, the addition of TAPI-1, an inhibitor of ADAMs, to the culture medium of cells without CHX treatment abolished the hypoxic loss of claudin-5. It is noteworthy that, in the presence of CHX, TAPI-1 rescued the endothelial cells from hypoxic loss of claudin-5 to the level of CHX-treated normoxic cells but not to the level of CHX-untreated normoxic cells (Fig. 2b,d). These findings indicate that some of ADAM family members accelerate the turnover of claudin-5 under hypoxic, not under normoxic, condition.

### Indispensable roles of ADAM12 and ADAM17 in the hypoxia-induced impairment of barrier property of endothelial cells

Among ADAM members with metalloproteinase activities, the expression of ADAMs 20, 28 and 33 could not be detected in bEnd.3 cells under normoxia (Fig. 2e), and therefore the siRNAs for ADAMs 8, 9, 10, 12, 15, 17, 19, 21 and 30 were introduced into the cultured endothelial cells to specify the responsible members. Specific as well as significant suppression of target molecules with the corresponding siRNAs was confirmed (Supplementary Fig. S1). Confocal microscopic images clearly showed that the hypoxia-induced disappearance of claudin-5 from cell membranes was abolished by the siRNAs targeted for either ADAM12 or ADAM17, while siRNAs for the other members showed no significant effects on the hypoxic changes in claudin-5 expression (Fig. 2f). TEERs of the cell monolayers, which were treated with the siRNAs for either ADAM12 or ADAM17, closely correlated with the corresponding immunofluorescence intensities of claudin-5 on cell membrane (Fig. 2g).

### Specificity of ADAM12 and ADAM17-mediated pathways for hypoxia-accelerated turnover of claudin-5

**Figure 3:**
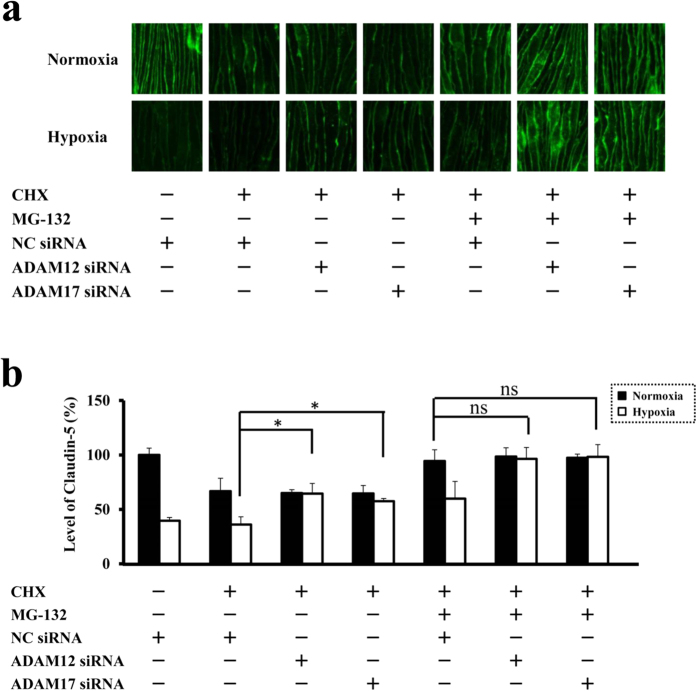
ADAM12 as well as ADAM17 are essential for the hypoxia-induced disappearance of claudin-5 from endothelial cell membranes. (**a** and **b**) Immunofluorescence images (**a**) and their corresponding quantitative analysis of claudin-5 levels on cell membranes (**b**) in bEnd.3 monolayers treated with CHX, MG-132, non-silencing siRNA (NC siRNA, for negative control), siRNA for ADAM12 and siRNA for ADAM17 as described. With CHX-treated cells, it is clearly shown that the suppression of either ADAM12 or ADAM17 expression with siRNA rescues the cells from the hypoxia-accelerated decrease in claudin-5 levels on cell membranes, while it has no significant effects on the oxygen concentration-independent decrease. Oxygen concentration-independent decrease in claudin-5 is abolished by inhibition of ubiquitin-proteasome system with MG-132. NC siRNA; non-silencing siRNA for negative control. *P < 0.05; ns, not significant.

In CHX-treated cells, the suppression of either ADAM12 or ADAM17 expression rescued the endothelial cells from hypoxic disappearance of claudin-5 to the level of CHX-treated normoxic cells, and the additional blockade of ubiquitin-proteasome with MG-132 led to the further increase in the claudin-5 immunofluorescence intensity to the level of normoxic cells without CHX treatment (Fig. 3a,b). These results clearly indicate the indispensable role of ADAM12 as well as ADAM17 in the hypoxia-induced disappearance of claudin-5 from cell membranes and consequently the impairment of neural vascular barrier function under hypoxia.

### Availability of ADAM12 and ADAM17 for therapeutic targets of hypoxia-induced impairment of neural vascular barrier

**Figure 4:**
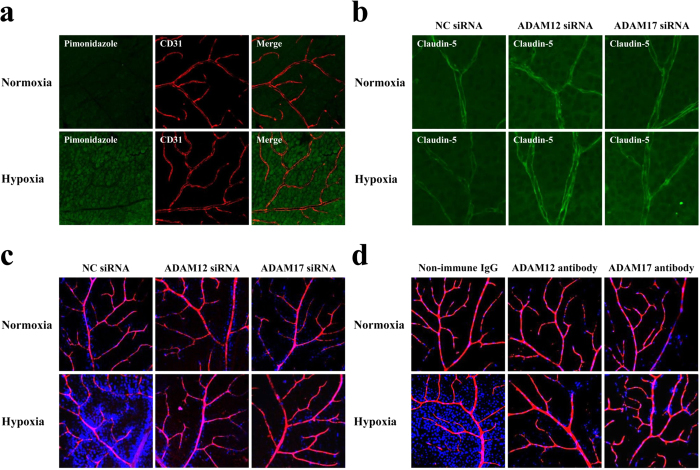
ADAM12 and ADAM17 are essential molecules for the impairment of barrier function of retinal vasculature under hypoxia. (**a**) Immunofluorescence images showing the oxygenation of retinas from mice under normoxia or hypoxia. Intraperitoneally injected pimonidazole hydrochloride (green) is incorporated in retinal cells from mice under hypoxia, as compared with minimal incorporation in retinal cells from mice under normoxia. Retinal vasculature is visualized by immunostaining for CD31 (red). (**b**) Immunofluorescence images, for claudin-5 expression, of flat mounts of retinas from mice under normoxia or hypoxia, with prior intravitreous injection of non-silencing siRNA (NC siRNA, for negative control), siRNA for ADAM12 or siRNA for ADAM17. Intravitreous injection of siRNA either for ADAM12 or ADAM17 rescues the retinal vasculature from the hypoxia-induced decrease in claudin-5 expression, in contrast to the hypoxic vasculature with the injection of non-silencing siRNA (NC siRNA). (**c** and **d**) Tracer experiment to evaluate the permeability of retinal vasculature under normoxia or hypoxia, with prior intravitreous injection of siRNAs (**c**) or neutralizing antibodies (**d**) specific for ADAM12 or ADAM17. For negative controls, non-silencing siRNA (NC siRNA) (c) and non-immune goat immunoglobulins (Non-immune IgG) (**d**) were injected intravitreously. Injected tracers, Hoechst stain (blue) and dextran (red), in flat mounts of retinas were detected under confocal microscopy, and merged views are presented. Nuclear staining of retinal cells by the extravasated Hoechst stain is noted in hypoxic retinas of negative controls, while the staining is minimal in hypoxic retinas with pre-injection of siRNAs or neutralizing antibodies for ADAM12 or ADAM17.

To validate *in vivo* the involvement of ADAM12 and ADAM17 in hypoxic disruption of neural vascular barrier, the permeability of retinal vasculature from mice kept under either normoxic (atmospheric air) or hypoxic (4 to 7% oxygen concentration) condition for 36 hours was monitored by intracardiac injection of fluorescence dyes, Hoechst stain H33258 (molecular mass, 534 Da) and tetramethylrhodamine-conjugated lysine-fixable dextran (molecular mass, 10,000 Da). Tissue hypoxia of retinas from mice kept under hypoxia was confirmed by increased incorporation of intraperitoneally injected pimonidazole hydrochloride (Hypoxyprobe-1) (Fig. 4a). In hypoxic retinas, the immunofluorescence signal of cell membrane-localized claudin-5 was diminished (Fig. 4b; left panels). In parallel with the decrease in claudin-5 expression, the vascular barrier function in hypoxic retinas was impaired, showing the increase in permeability against a smaller molecule Hoechst stain H33258 (Fig. 4c,d; left panels). It is noteworthy that the prior injection of ADAM12-specific or ADAM17-specific siRNA into the vitreous cavities clearly rescued the retinal vasculature from the decrease in claudin-5 expression as well as the enhanced leakage of dye (Fig. 4b,c; center and right panels). The same inhibitory effect on hypoxia-induced impairment of vascular barrier was obtained with the prior intravitreous injection of a neutralizing antibody against either ADAM12 or ADAM17 (Fig. 4d; center and right panels). These *in vivo* findings support the *in vitro* data demonstrating the indispensable roles of ADAM12 and ADAM17 in hypoxia-induced impairment of neural vascular barrier function, and furthermore indicate the availability of ADAM12 and ADAM17 for therapeutic targets in the intractable neural diseases with vascular barrier dysfunction.

## Discussion

In the present study, we have elucidated an aspect of regulatory mechanisms of neural vascular barrier by demonstrating for the first time that ADAM12 and ADAM17 in endothelial cells are the essential molecules for hypoxia-induced impairment of vascular barrier function. ADAM12 was originally identified as a transmembrane molecule involved in the fusion of muscle cells, and initially named meltrin α^18^,^19^. A series of studies have highlighted the role of ADAM12 in pathological situations, especially in cancer, through processing molecules including insulin-like growth factor-binding protein (IGFBP)-3, IGFBP-5 and heparin-binding epidermal growth factor^20^,^21^,^22^. Related to the neural vascular barrier, ADAM12 expressed in T cells was suggested to potentiate the transmigration of T cells through vascular barrier in the experimental autoimmune encephalomyelitis mice^23^. As concerns the vascular cells, ADAM12 induced in endothelial cells of tumor vessels was suggested to be involved in tumor progression of breast invasive ductal carcinoma through processing vascular endothelial cadherin and Tie-2^24^. ADAM17, another essential molecule identified in this study, was originally reported as an enzyme responsible for cleavage of transmembrane precursor of tumor necrosis factor α (TNF-α) to release the active TNF-α, and therefore called TNF-α converting enzyme (TACE)^25^,^26^. Later on, ADAM17 has turned out to play the roles in a wide range of physiological and pathological situations by processing the membrane-bound molecules such as epidermal growth factor receptor ligands^27^ and JAM^13^. Cleavage of endothelial cell membrane-bound JAM by ADAM17 is reported to suppress the transendothelial migration of neutrophils^13^. Our present data is the first to demonstrate that ADAM12 as well as ADAM17 are involved in the regulation of neural vascular barrier function, and furthermore their involvement is oxygen concentration-dependent.

ADAM family members are known to process the target membrane-bound molecules through the quick induction of their protease activities under interaction with other molecules. Therefore, it can be true that ADAM12 and ADAM17 which are expressed, already under normoxia, in neural vascular endothelial cells enable the endothelial cells to change their barrier properties in a way of quick response to the decrease in environmental oxygen concentration. In fact, the subcellular localization of ADAM12 as well as ADAM17 in bEnd.3 cells was found to be changed promptly from cytoplasmic to cell membrane-associated within 30 minutes after hypoxic stimulation (Supplementary Fig. S2). Thus, ADAM12 and ADM17 can be potentiated to process the cell membrane-associated molecules in quick response to hypoxia. It remains possible that the other molecules are involved in the hypoxic impairment of neural vascular barrier, especially in chronic phase under hypoxia. However, the pivotal roles of ADAM12 as well as ADAM17 also in chronic phase of hypoxia are demonstrated by the findings that the *in vivo* retinal vasculature under chronic hypoxia was successfully rescued from barrier impairment by intravitreous injection of the siRNA or neutralizing antibody targeting either ADAM12 or ADAM17. Their critical roles in barrier impairment under chronic hypoxia is also supported by the *in vitro* data that the suppression of either ADAM12 or ADAM17 could abolished the loss of the barrier properties not only in cultured endothelial cells under hypoxia for 30 minutes but in cells under hypoxia for 24 hours (data not shown). These results indicate the potential of ADAM12 and ADAM17 for the targets to establish new therapies of intractable neural with hypoxia-induced impairment of vascular barrier.

In various pathological situations, including diabetic retinopathy and ischemic cerebral attack, the hypoxia of affected neural tissues is known to accelerate the disease progression through impairment of vascular barrier^1^,^6^,^7^. Dysfunction of vascular barrier in hypoxic retina reduces the visual acuity of patients with diabetic retinopathy, and therefore the restoration of vascular barrier function would improve the visual acuity of patients. In patients with ischemic cerebral attack, the rescue of neural cells in ischemic penumbra, which is a hypoxic area around the ischemic core and still contains viable cells, from going to necrosis must be focused on to improve the neurological outcome of patients. Thus, the modulation of neural vascular barrier function in neural tissues under pathological hypoxia is considered to be a promising therapeutic strategy to control the subjective as well as objective symptoms of patients with intractable neural diseases. In the present study, we have successfully specified the molecules which can be the therapeutic targets to restore the vascular barrier function in hypoxic neural tissues. These molecules are not only the potential therapeutic targets for neural diseases with hypoxia-induced impairment of vascular barrier, but also provide a clue to the identification of molecules which would be involved in the vascular barrier dysfunction due to various triggers and consequently would be the targets to establish new therapies applicable to a wide range of intractable neural diseases with vascular barrier impairment.

## Methods

### Cell Culture

A mouse brain microvascular endothelial cell line, bEnd.3, was purchased from the American Type Culture Collection (Manassas, VA), and cultured in Dulbecco’s Modified Eagle’s medium with 4500 mg/l glucose (Sigma-Aldrich, St. Louis, MO) supplemented with 10% fetal bovine serum, at 37 °C in a humidified incubators either with 5% CO2 and 95% atmospheric air for normoxia or with 5% CO_2_ and 1%O_2_ balanced with N_2_ for hypoxia. Oxygen-regulated Personal Multi Gas incubator (Astec Co., Ltd., Tokyo, Japan) was used to generate the hypoxic culture condition. Cycloheximide (CHX; 200 μg/ml; Sigma-Aldrich), MG-132 (50 μM; Sigma-Aldrich) and TAPI-1 (100 μM; Enzo Life Sciences, Farmingdale, NY) were added to culture medium, in order to inhibit the protein synthesis, the degradation by ubiquitin-proteasome system and the proteinase activities of ADAMs, respectively.

### Immunofluorescence Staining of Cultured Cells

bEnd.3 monolayers were fixed with 100% methanol for 5 minutes at room temperature and were incubated with 10% Non-Immune Goat Serum (Invitrogen, Carlsbad, CA) for 30 minutes to block the nonspecific binding of antibodies. Then, the cell monolayers were reacted with rabbit polyclonal antibody against claudin-5 (1/25 dilution; Invitrogen), goat polyclonal antibody against ADAM12 (1/200 dilution; C-20, sc-16527; Santa Cruz Biotechnology, CA), or goat polyclonal antibody against ADAM17 (1/200 dilution; C-15, sc-6416; Santa Cruz Biotechnology, CA) at 4°C overnight. After washing in PBS, the cell monolayers were incubated with Alexa Fluor 488 goat anti-rabbit IgG (1/200 dilution; Molecular Probes, Eugene, OR) for the staining of claudin-5, or Alexa Fluor 488 donkey anti-goat IgG (1/200 dilution; Molecular Probes, Eugene, OR) for the staining of ADAM12 and ADAM17 at room temperature under light protection for 1 hour. Stained cell monolayers were mounted in Fluorescence Mounting Medium (Dako Denmark A/S, Glostrup, Denmark), and observed under a Zeiss LSM5 Pascal laser confocal microscope and a Zeiss Axio Observer.Z1 fluorescence microscope (Carl Zeiss, Jena, Germany). For a quantitative analysis, the fluorescence intensities of claudin-5 on plasma membranes were measured using an operation menu installed in LSM5 Pascal. Three fields of a cell monolayer were randomly photographed, and 3 straight lines were drawn on each photograph. Then, fluorescence intensities at the points of cell membranes intersected with drawn straight lines were quantified. The mean value of fluorescence intensities, at around 80 points, was calculated as the level of claudin-5 on cell membrane for each monolayer. All experiments were performed independently in triplicate.

### Transendothelial Electrical Resistance (TEER)

Electrical resistance across a bEnd.3 monolayer was measured as described previously^9^. In brief, bEnd.3 cells were grown on fibronectin-coated inserts of 0.4 mm pore size to the confluence, and the electrical resistance of the inserts was measured with the Millicell ERS Voltohmmeter (Millipore, Billerica, MA). TEERs of bEnd.3 monolayers were calculated by subtracting the resistance of blank inserts without cells and multiplying the subtracted values by the surface areas of inserts. Each experiment was performed in triplicate.

### Reverse Transcription-Polymerase Chain Reaction (RT-PCR)

To determine the mRNA levels of ADAMs 8, 9, 10, 12, 15, 17, 19, 20, 21, 28, 30 and 33, total RNA was isolated from bEnd.3 cells using RNeasy Plus Mini Kit (Qiagen Inc., Chatsworth, CA). Total RNA (5 μg) was reverse-transcribed in a 33 μl reaction mixture with StrataScript First Strand Synthesis System (GE Healthcare, Buckinghamshire, UK) according to manufacturer’s instructions, and 2 μl of the reaction mixture was subjected to PCR amplification of ADAMs 8, 9, 10, 12, 15, 17, 19, 20, 21, 28, 30 and 33, and GAPDH (internal control). Nucleotide sequences of primers for PCR amplification were listed in Supplementary Table S1. PCR was performed in a 50 μl reaction mixture containing 100 μM dNTPs, 800 nM each primer and 5 units of TaKaRa Ex Taq polymerase (TAKARA BIOTECNOLOGY, Otsu, Shiga, Japan), and. The PCRs of ADAMs 8, 10, 12 and 17 were performed using the following conditions: initial denaturation at 95 °C for 5 min; 30 amplification cycles (denaturation at 95 °C for 30 seconds, annealing at 60 °C for 30 seconds and extension at 72 °C for 30 seconds); and a final extension at 72 °C for 10 minutes. For ADAMs 9, 15 and 33 and GAPDH, the PCR condition was the same as above except for the annealing temperature used: for ADAM9, the annealing temperature was 50 °C, whereas for ADAMs 15, 33 as well as GAPDH it was 55 °C. The PCRs of ADAMs 19, 20, 21, 28 and 30 were performed using the following conditions; initial denaturation at 95 °C for 5 min, 30 amplification cycles (denaturation at 94 °C for 60 seconds, annealing at 60 °C for 60 s and extension at 72 °C for 90 seconds) and a final extension at 72 °C for 10 minutes. The PCR products were analyzed by using Agilent 2100 Bioanalyzer (Agilent Technologies, Palo Alto, CA).

### Transfection of small interfering RNA (siRNA) and Real-Time Quantitative Polymerase Chain Reaction (Real-Time PCR)

Non-silencing siRNA for negative control and siRNAs specific for ADAM family members as well as GAPDH were purchased from Applied Biosystems (Applied Biosystems, Foster City, CA), and their IDs are listed in Supplementary Fig. 1. For bEnd.3 cells in each well of 24-well plate, 50 μl of 0.24 μM siRNA solution diluted in Gibco Opti-MEM I (Life Technologies. Paisley, UK) was gently mixed with 50 μl of Gibco Opti-MEM I containing 1μl of Lipofectamine RNAiMAX (Invitrogen, Carlsbad, CA). After further incubation at room temperature for 20 minutes, 100 μl of the reaction mixture was added to each well with 500 μl of culture medium (final concentration of siRNA; 20 nM). After 48 hours, the cells were processed for experiments. Specific and significant silencing of target molecules was analyzed by Real-Time PCR according to the manufacture’s instruction. Total RNA was isolated from bEnd.3 cells, reverse-transcribed and subjected to the Real-Time PCR reaction with the Eagle Taq Master Mix Kit (Roche Molecular Systems, Branchburg, NJ) and with TaqMan Gene Expression Assays (Applied Biosystems). IDs of TaqMan Gene Expression Assays used in this study are listed in Supplementary Fig. 1. Reaction in 96-well plate was performed with StepOnePlus™ Real-Time PCR System (Applied Biosystems). The thermal cycle conditions were as follows; 2 min at 50 °C and 10 min at 95 °C, followed by 40 cycles at 95 °C for 15 s and 60 °C for 60 s. The comparative C_T_ (ΔΔC_T_) method of relative quantification was used to determine the fold change in gene expression. With the comparative C_T_ method, the StepOne software measures amplification of the target as well as the endogenous control (β-actin) in a sample. Real-Time PCR was repeated in triplicate for each sample.

### Animal Studies

This research was approved by the Institutional Animal Care and Use Committee (IACUC) in Yamaguchi University, and carried out under the control of the Rule for the Care and Use of Laboratory Animals in Yamaguchi University and The Law (No.105), Notification (No.88) and Guideline (No.71) of the Government.

Male C57BL/6J mouse (7 week old; Japan SLC, Shizuoka, Japan) were used in this study. Mice were maintained for 36 hours in a chamber in which the oxygen concentration could be regulated by controlling the inflow rates of nitrogen^9^. For hypoxic condition, the oxygen concentration was maintained at 4 to 7% by continuous monitoring with Oxygen Monitor JKO-25 Version II series (JIKCO LTD, Tokyo, Japan). For normoxic condition, mice were kept in atmospheric air. To analyze the retinal vasculature, the eyes were enucleated, independently by M. A., immediately after taking mice out of chambers, and flat mounts of retinas were prepared by D. C. for immunohistochemistry or direct observation under microscope. Each experiment was repeated at least 3 times to confirm the reproducibility of results.

### Detection of Hypoxia in Retinal Tissues

Tissue hypoxia in mouse retinas was evaluated using the Hypoxyprobe Plus Kit (Hypoxyprobe, Burlington, MA). Pimonidazole hydrochloride was administered intraperitoneally to mice at a dosage of 60 mg/kg body weight. The mice were euthanized 40 minutes after the injection of pimonidazole hydrochloride, and retinal flat mounts were prepared. After the fixation in 4% paraformaldehyde (PFA), retinal flat mounts were incubated with both the FITC conjugated mouse anti-pimonidazole monoclonal antibody (1/100 dilution) and the rat monoclonal antibody against CD31 (PECAM-1) (1/100 dilution; BD Biosciences, San Jose, CA) at 4 °C overnight, and subsequently reacted with Alexa Fluor 546 goat anti-rat IgG (1/200 dilution; Molecular Probes) at room temperature for 3 hours. An antibody against CD31 was used to visualize retinal vasculature by staining of endothelial cells. The stained flat mounts were observed with a Zeiss LSM 510 META laser confocal microscope Carl Zeiss.

### Immunofluorescence Staining of Retinal Flat Mounts

Flat mounts of retinas were fixed with 4% PFA at room temperature for 30 minutes, and were treated with PBS containing 1% BSA and 0.5% Triton X-100 at room temperature for 1 hour for blocking the nonspecific binding of antibodies as well as the permeabilization of tissues. Subsequently, the flat mounts were reacted with rabbit polyclonal antibody against claudin-5 (1/25 dilution; Invitrogen) at 4 °C overnight. After washing with PBS containing 0.1% Tween20, they were incubated with Alexa Fluor 488 goat anti-rabbit IgG (1/200 dilution; Molecular Probes) at room temperature for 3 hours. The retinal flat mounts were then washed with PBS and mounted in fluorescent mounting medium for observation under a Zeiss LSM5 Pascal laser confocal microscope (Carl Zeiss).

### Intravitreous Injection of siRNAs and Neutralizing Antibodies

siRNAs or antibodies against ADAM12 and ADAM17 were injected into mouse vitreous cavities to suppress their expressions or to neutralize their functions, respectively. For administration of siRNAs, 1 μl of 50 nM siRNA solution was combined with 1 μl of Lipofectamine RNAiMAX (Invitrogen) in 8 μl of Gibco Opti-MEM I (Life Technologies). After the incubation at room temperature for 20 minutes, 1 μl of the reaction mixture was injected into vitreous cavities of mice under anesthesia with pentobarbital sodium (65 mg/kg body weight). A siRNA targeting ADAM12 or ADAM17 was injected into an eye of a mouse, and the non-silencing siRNA (NC siRNA, for negative control) was injected into the other eye. For administration of antibodies with neutralizing activities, the goat polyclonal antibody against ADAM12 (C-20, sc-16527) and the goat polyclonal antibody against ADAM17 (C-15, sc-6416) were purchased from Santa Cruz Biotechnology. 0.2 μg of the antibody was injected into an eye of a mouse under anesthesia, while non-immune goat immunoglobulins from goat serum (Non-immune IgG) (Sigma-Aldrich) was injected into the other eye. A 10-μl Hamilton syringe with a 32-gauge needle (Hamilton Company, Reno, NV) was used for intravitreous injection of siRNAs or antibodies. Mice had been subjected to normoxic or hypoxic condition from 12 to 48 hours after the injection of siRNAs or antibodies, and retinal flat mounts were prepared for the immunohistochemistry or the permeability assay of retinal vasculature.

### Permeability Assay of Retinal Vasculature

To evaluate the vascular permeability of retinal tissues, 500 μl of saline containing 100 μg/ml Hoechst stain H33258 (molecular mass, 534 Da; Sigma-Aldrich) and 1 mg/ml tetramethylrhodamine-conjugated lysine-fixable dextran (molecular mass, 10 kDa; Molecular Probes) was injected into the left ventricle^9^,^28^. The tracers were allowed to circulate for 5 minutes, and eyes were enucleated and immediately fixed in 4% PFA for 30 minutes. Then, retinal flat mounts were prepared and observed under a Zeiss LSM510 META laser confocal microscope (Carl Zeiss). In a mouse under hypoxia, the hypoxia-induced impairment of vascular barrier was confirmed by leakage of dye from retinal vasculature in an eye treated with NC siRNA or non-immune IgG.

### Statistical Analyses

Data were statistically analyzed with Student’s *t*-test, since the variance was shown to be equal with F-test between the groups which were to be compared in this study. Differences were considered to be statistically significant at *P *< 0.05. All data are presented as mean ± SD.

## Additional Information

**How to cite this article**: Cui, D. *et al.* ADAM12 and ADAM17 are essential molecules for hypoxia-induced impairment of neural vascular barrier function. *Sci. Rep.* **5**, 12796; doi: 10.1038/srep12796 (2015).

## Supplementary Material

Supplementary Information
