# Supplementary material for: ADAM12 and ADAM17 are essential molecules for hypoxia-induced impairment of neural vascular barrier function
Source: Sci Rep. 2015 Aug 5;5:12796. doi: 10.1038/srep12796 (PMC4525292; doi:10.1038/srep12796)
Supplement: Supplementary Information [file srep12796-s1.pdf]

Supplementary Information

(Supplementary Figure S1, Supplementary Figure S2 and Supplementary Table S1)

ADAM12 and ADAM17 are essential molecules for hypoxia-induced impairment of neural vascular barrier function

Dan Cui[1], Mitsuru Arima[1,2], Keiyo Takubo[3,4], Tokuhiro Kimura[1], Keisuke Horiuchi[5], Takuya Minagawa[6], Satoshi Matsuda[7], Eiji Ikeda[1]

[1]Department of Pathology, Yamaguchi University Graduate School of Medicine, 1-1-1 Minami-Kogushi, Ube, Yamaguchi 755-8505, Japan; [2]Department of Ophthalmology, Kyushu University Graduate School of Medical Sciences, 3-1-1 Maidashi, Higashi-ku, Fukuoka City, Fukuoka 812-8582, Japan; [3]Department of Stem Cell Biology, Research Institute, National Center for Global Health and Medicine, 1-21-1 Toyama, Shinjuku-ku, Tokyo 162-8655, Japan; Departments of [4]Cell Differentiation, The Sakaguchi Laboratory of Developmental Biology, [5]Orthopedic Surgery and [6]Surgery, Keio University School of Medicine, 35 Shinanomachi, Shinjuku-ku, Tokyo 160-8582, Japan; [7]Department of Cell Signaling, Institute of Biomedical Sciences, Kansai Medical University, 10-15 Fumizono-cho, Moriguchi, Osaka 570-8506, Japan.

a

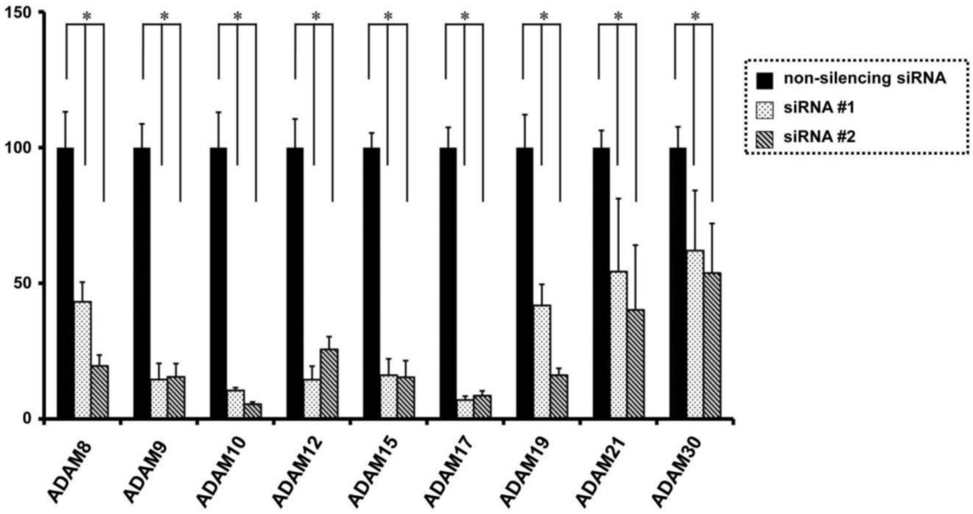

b

Gene Name	siRNA ID	TaqMan Gene Expression Assays ID
Adam 8	s61982	Mm00545762_m1
	s61983	
Adam 9	s232235	Mm01218460_m1
	s232236	
Adam 10	s61947	Mm00545742_m1
	s61945	
Adam 12	s61951	Mm00475719_m1
	s61952	
Adam 15	s61954	Mm00477328_m1
	s61955	
Adam 17	s61959	Mm00456428_m1
	s61958	
Adam 19	s61961	Mm00477337_m1
	s61962	
Adam 21	s80809	Mm00480375_s1
	s80808	
Adam 30	s89401	Mm00810549_s1
	s89401	

Supplementary Figure S1: Silencing of ADAM family members with siRNAs.

a, Quantitative analysis of RNAs of targeted ADAM family members. Two kinds of siRNAs were designed for each member, and their significant silencing effects are presented. b, IDs of siRNAs and TaqMan Gene Expression Assays targeting ADAM family members. Information about these IDs can be obtained on the homepage of Applied Biosystems.

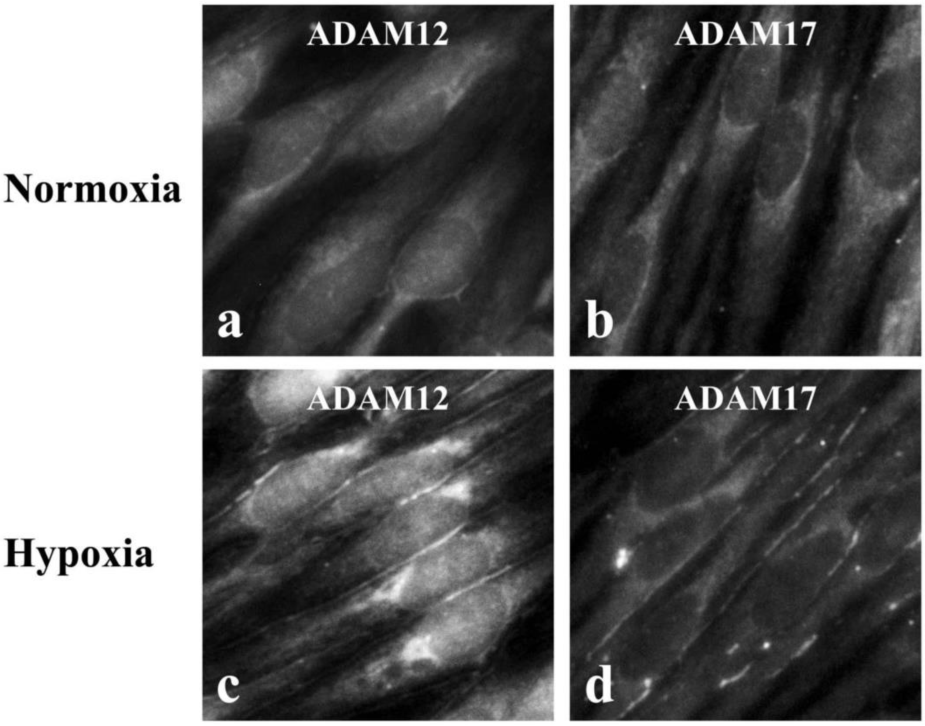

Supplementary Figure S2: Subcellular localization of ADAM12 and ADAM17 in endothelial cells under normoxia and hypoxia. Immunofluorescence images for the subcellular localization of ADAM12 (a, c) and ADAM17 (b, d) in bEnd.3 cells under normoxia (a, b) and hypoxia (c, d) are presented. Cell membrane-associated localization of ADAM12 as well as ADAM17 in bEnd.3 cells is enhanced under hypoxia. Specificities of fluorescence signals for ADAM12 and ADAM17 were confirmed by the abolishment of signals in cells pretreated with siRNAs for ADAM12 and ADAM17, respectively (data not shown).

Supplementary Table S1 | Nucleotide sequences of primers for PCR

		Primer sequence	Product size
ADAM8	Forward	5′-TTGCCCCATGTGAAACAGTA-3′	408 bp
	Reverse	5′-GATGTTTGCCTGATACATCGC-3′	
ADAM9	Forward	5′-TTGCTCATGAATTGGGGCATAAC-3′	425 bp
	Reverse	5′-CAGTACTCAGGAACATCACA-3′	
ADAM10	Forward	5′-CCATCAACTTGTGCCAGTAC-3′	421 bp
	Reverse	5′-CCCATTTGATAACTCTCTCG-3′	
ADAM12	Forward	5′-CTTGACTGTAGGAATCCTGG-3′	494 bp
	Reverse	5′-CTCACCAAGGCACTAGTGAG-3′	
ADAM15	Forward	5′-GGAGAGCAGTGTGACTGTGGC-3′	186 bp
	Reverse	5′-GCAGAACTCAGGCAGATCACA-3′	
ADAM17	Forward	5′-CACTTTTGGGAAGTTTCTGG-3′	492 bp
	Reverse	5′-CTCTGTCTCTTTGCTGTCAAC-3′	
ADAM19	Forward	5′-GGCTGGTGATGACTGGAA-3′	429 bp
	Reverse	5′-GCTTGAAAGTTGGGTTGG-3′	
ADAM20	Forward	5′-CTGATAGAGCATTCTACAGTGC-3′	608 bp
	Reverse	5′-TGCTGTGATAGCTAATGCTT-3′	
ADAM21	Forward	5′-TCTGGCTTGGGGTATTTTTG-3′	500 bp
	Reverse	5′-TTGGCGTGCTACTTCCTTCT-3′	
ADAM28	Forward	5′-GATGGGATGGTTCAAGAACC-3′	644 bp
	Reverse	5′-TGGTCCTGAACAATGCCAAC-3′	
ADAM30	Forward	5′-AACCAGGTGCCAACTGTAGC-3′	496 bp
	Reverse	5′-CCCATGGGTTTCATGGATAG-3′	
ADAM33	Forward	5′-CTGAGAACTCTCATGAGC-3′	540 bp
	Reverse	5′-GACTCAGTCGAACCATAG-3′	
GAPDH	Forward	5′-TGAAGGTCGGAGTCAACGGATTTGGT-3′	930 bp
	Reverse	5′-CATGTGGGCCATGCGGTCCACCAC-3′	
